# Giant desmoplastic cutaneous squamous cell carcinoma of the gluteal region

**DOI:** 10.1186/s12957-017-1191-7

**Published:** 2017-07-04

**Authors:** Darko Katalinic, Antonio Juretic

**Affiliations:** 1 Department of Internal Medicine, Faculty of Medicine, J.J. Strossmayer University of Osijek, Cara Hadrijana 10/E, HR-31000 Osijek, Croatia; 2 Department of Oncology,Faculty of Medicine, University Hospital Centre Zagreb, University of Zagreb, Zagreb, Croatia

**Keywords:** Cutaneous squamous cell carcinoma, Histology, Therapy

## Abstract

Cutaneous squamous cell carcinoma (cSCC) is the most common type of skin tumour with the ability of metastatic spread. It represents about 20% of all malignancies diagnosed worldwide each year. Despite increased knowledge regarding the causes of skin cancer, the incidence of cSCC rises. The disease originates from epidermal keratinocytes, but it may occur on all areas of the body. It has an invasive nature and the potential to metastasise. We report unusual case of a giant metastatic desmoplastic cSCC of the gluteal region in a patient with previously resected desmoplastic cSCC presenting 8 months later with multiple liver and lung metastases.

To the Editor,

A 55-year-old man presented with gradually enlarging pigmented skin lesion since many months. The lesion was located on his left hip measuring 5 × 5 cm and was surgically excised with a free margin of healthy tissue. Histopathological examination of the incisional biopsy showed the entire thickness of the epithelium filled with atypical CK5+, TTF−, CK7− and napsin A− keratinocytes. The tumour cells also demonstrated hyperchromasia, nuclear pleomorphism and increased number of mitosis, all consistent with diagnosis of desmoplastic cutaneous squamous cell carcinoma (cSCC) (Fig. 1a). The patient underwent regular examinations, and his condition remained stable for 8 months. After that period, he presented with growing, inflamed mass in the gluteal region. The metastatic work-up was positive and confirmed a diagnosis of stage IV desmoplastic cSCC with liver and lung metastases (Fig. 1b, c). Clinical and laboratory examinations did not reveal any signs of depleted immune system. The patient refused further treatment and unfortunately died after 2 months as a result of tumour progression (Fig. 1d, e). The ultimate cause of death was acute hepatic failure due to organ invasion by neoplastic cells.

**Fig. 1:**
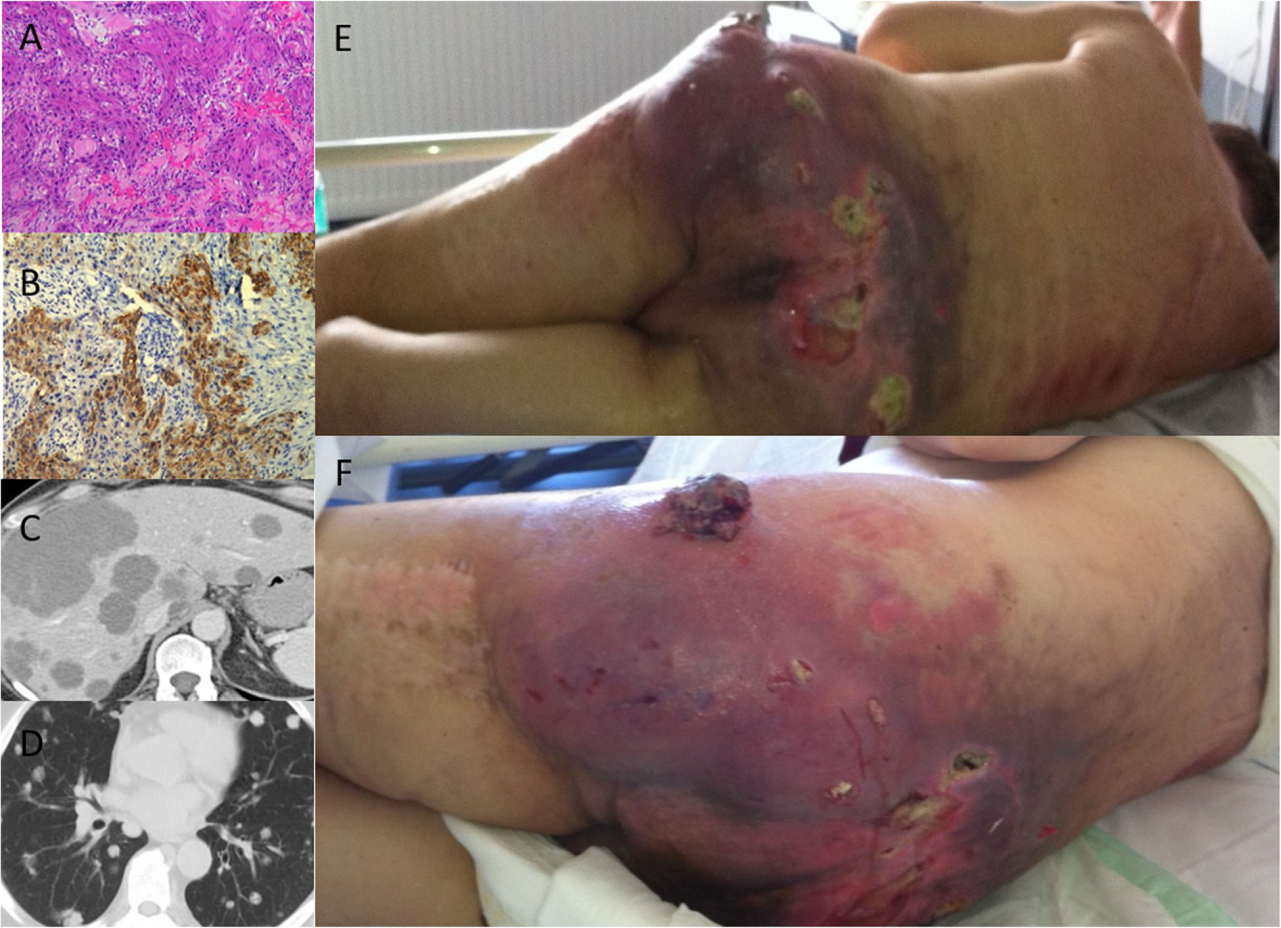
Histopathological analysis showing desmoplastic cutaneous squamous cell carcinoma (**a** HE staging, original magnification ×200; **b** cytokeratin AE1/AE3 immunostaining, original magnification ×200). Radiological evaluation revealed a metastatic involvement of the lung and the liver (**c**, **d**). Clinical picture of the exulcerated, inflamed bleeding desmoplastic cutaneous squamous cell carcinoma of the *lef*t hip (**e**, **f**)

cSCC is the second most common form of non-melanoma skin cancers and accounts for approximately 20% of all cutaneous malignancies. cSCC occurs in men two to three times more frequently than it does in women. Its global incidence varies geographically, from 0.03 to 3.5 cases per 100,000 people per year and seems to have increased over the past 30 years by 50 and up to 200% [1, 2]. In contrast to basal cell carcinoma, cSCC is aggressive malignancy and easily metastasise via hematogenous and lymphogenous routes. cSCC usually occurs on the upper portions of the body, and the main symptom is a wart-like growth that may have a rough, scaly surface with flat reddish patches which occasionally bleeds or it has elevated growth with central depression that persists for weeks and may rapidly increase in size. After the treatment, the overall prognosis for the majority of patients is excellent with an overall 5-year cure rate of greater than 90 and 4.6% rate of recurrence [1, 2]. The metastatic potential has been estimated to range from 2 to 5%, but this estimation should be considered with caution. The presence of distant metastatic disease is associated with median survival of less than 2 years [1, 2]. The most prominent risk factors include exposure to sun or artificial ultraviolet radiation, leukoplakia, Bowen’s disease, infection with human papillomavirus, genetic factors such as mutations in the MC1R gene, genetic defects in DNA repair, therapy with immunosuppressive agents and advanced age [3–5]. Mutations in the suppressor gene p53 and RAS gene as well as activation of EGFR are the most common genetic abnormalities found in cSCCs [2, 4–6]. The differential diagnosis of cSCC may include keratoacanthoma, basal cell carcinoma, amelanotic melanoma, fibroxanthoma, pseudoepitheliomatous hyperplasia or HPV-induced papillomas [2]. Histopathologically, the subtypes of cSCC include verrucous, spindle, desmoplastic, acantholytic and adenosquamous form [2]. A biopsy or excision of the lesion and the histologic examination including immunohistochemistry, especially cytokeratin AE1/AE3 immunostaining and 3D histology, should be performed in all clinically suspicious lesions [7].

The cSCC is generally treated by surgical excision, electrodessication and curettage. Non-surgical options for the treatment include cryotherapy, topical chemotherapy, photodynamic therapy, topical immune response modifiers, radiotherapy and systemic chemotherapy [2]. In the present case, the patient was initially diagnosed with desmoplastic cSCC and treated with surgical resection in combination with plastic reconstruction with preservation of function and satisfactory cosmetic results. The remission lasted 8 months. After that period, he was diagnosed with relapsed, poorly differentiated stage IV desmoplastic cSCC which is clinically characterised by a highly infiltrative growth and high metastatic potential (Fig. 1d, e). The probable cause for relapse in this case was incomplete clearence of the micrometastatic cancer cells that surrounded the primary tumour. The palliative radiotherapy and chemotherapy were not indicated according to patient’s own conditions and wishes. However, stage IV cSCC is responsive to a variety of different chemotherapeutic agents, but there is no established standard regimen. Among most common non-targeted agents used are platin salts, 5-fluorouracil or taxanes [2, 8, 9]. Polychemotherapy should be reserved for patients requiring more aggressive management, while otherwise, mono-chemotherapy should be considered as a first-line treatment [2, 8, 9]. A chimeric immunoglobulin G1 monoclonal antibody cetuximab has been reported in the literature as a successful second-line option [9, 10]. Some tyrosine kinase inhibitors (gefitinib, dasatinib, erlotinib) as well as different immunotherapy agents (anti-PD1 antibody, pembrolizumab) demonstrated encouraging results in the treatment of cSCC [11–16].

Finally, it is estimated that majority of all cSCC recurrences will develop within 2 years of the initial diagnosis [17]. Therefore, all patients with cSCC should be followed closely with regular follow-up schedule such as every 6 months depending on aggressiveness of the tumour and clinical and histological criteria.

## Abbreviations

CK5: Cytokeratin 5
CK7: Cytokeratin 7
cSCC: Cutaneous squamous cell carcinoma
DNA: Deoxyribonucleic acid
EGFR: Epidermal growth factor receptor
HPV: Human papillomavirus
MC1R: Melanocortin-1 receptor gene
p53: Tumour suppressor gene p53
RAS: Ras-protooncogene
TTF: Thyroid transcription factor
